# Hairy Transcriptional Repression Targets and Cofactor Recruitment in *Drosophila*

**DOI:** 10.1371/journal.pbio.0020178

**Published:** 2004-07-13

**Authors:** Daniella Bianchi-Frias, Amir Orian, Jeffrey J Delrow, Julio Vazquez, Alicia E Rosales-Nieves, Susan M Parkhurst

**Affiliations:** **1**Division of Basic Sciences, Fred Hutchinson Cancer Research CenterSeattle, Washington, United States of America; **2**Genomics Resource, Fred Hutchinson Cancer Research CenterSeattle, Washington, United States of America; **3**Scientific Imaging, Fred Hutchinson Cancer Research CenterSeattle, WashingtonUnited States of America

## Abstract

Members of the widely conserved Hairy/Enhancer of split family of basic Helix-Loop-Helix repressors are essential for proper *Drosophila* and vertebrate development and are misregulated in many cancers. While a major step forward in understanding the molecular mechanism(s) surrounding Hairy-mediated repression was made with the identification of Groucho, *Drosophila* C-terminal binding protein (dCtBP), and *Drosophila* silent information regulator 2 (dSir2) as Hairy transcriptional cofactors, the identity of Hairy target genes and the rules governing cofactor recruitment are relatively unknown. We have used the chromatin profiling method DamID to perform a global and systematic search for direct transcriptional targets for *Drosophila* Hairy and the genomic recruitment sites for three of its cofactors: Groucho, dCtBP, and dSir2. Each of the proteins was tethered to Escherichia coli DNA adenine methyltransferase, permitting methylation proximal to in vivo binding sites in both *Drosophila* Kc cells and early embryos. This approach identified 40 novel genomic targets for Hairy in Kc cells, as well as 155 loci recruiting Groucho, 107 loci recruiting dSir2, and wide genomic binding of dCtBP to 496 loci. We also adapted DamID profiling such that we could use tightly gated collections of embryos (2–6 h) and found 20 Hairy targets related to early embryogenesis. As expected of direct targets, all of the putative Hairy target genes tested show Hairy-dependent expression and have conserved consensus C-box–containing sequences that are directly bound by Hairy in vitro. The distribution of Hairy targets in both the Kc cell and embryo DamID experiments corresponds to Hairy binding sites in vivo on polytene chromosomes. Similarly, the distributions of loci recruiting each of Hairy's cofactors are detected as cofactor binding sites in vivo on polytene chromosomes. We have identified 59 putative transcriptional targets of Hairy. In addition to finding putative targets for Hairy in segmentation, we find groups of targets suggesting roles for Hairy in cell cycle, cell growth, and morphogenesis, processes that must be coordinately regulated with pattern formation. Examining the recruitment of Hairy's three characterized cofactors to their putative target genes revealed that cofactor recruitment is context-dependent. While Groucho is frequently considered to be the primary Hairy cofactor, we find here that it is associated with only a minority of Hairy targets. The majority of Hairy targets are associated with the presence of a combination of dCtBP and dSir2. Thus, the DamID chromatin profiling technique provides a systematic means of identifying transcriptional target genes and of obtaining a global view of cofactor recruitment requirements during development.

## Abstract 2

A new chromatin profiling technique identifies targets of the transcription factor Hairy -- as well as targets of three co- repressors -- which implicate Hairy in several developmental processes

## Introduction

Transcriptional repression is an important feature of developmental processes, where it is necessary for establishing intricate patterns of gene expression (reviewed in Herschbach and Johnson 1993; Gray and Levine 1996; Hanna-Rose and Hansen 1996; Courey and Jia 2001; Gaston and Jayaraman 2003). *Drosophila* embryogenesis is marked by the subdivision of the embryo into progressively more precise spatial domains, achieved through the coordinated functions of both transcriptional activators and repressors (maternal→gap→pair-rule→segment polarity; for review, see Lawrence 1992). One such developmental repressor is the pair-rule gene *hairy,* which sits at a key position in the segmentation gene hierarchy: it is one of the first genes to show the reiterated periodicity that is central to the establishment of proper embryonic body plan throughout metazoa (Ingham et al. 1985).

During segmentation, *hairy* behaves genetically as a negative regulator of a downstream (secondary) pair-rule gene, *fushi tarazu* (*ftz*; Carroll and Scott 1986; Howard and Ingham 1986). In addition to embryonic segmentation, Hairy also regulates several other developmental processes (cf. Brown et al. 1995; Davis and Turner 2001; Myat and Andrew 2002). For example, during larval development, Hairy is required for proper peripheral nervous system development, where it is a negative regulator of the proneural basic Helix-Loop-Helix (bHLH) activator gene *achaete* (*ac;* Botas et al. 1982; Ohsako et al. 1994; Van Doren et al. 1994).

Hairy belongs to the evolutionarily conserved Hairy/Enhancer of split/Deadpan (HES) subclass of repressor bHLH proteins (Rushlow et al. 1989). These proteins function throughout development as dedicated transcriptional repressors of genes necessary for cell fate decisions in processes including segmentation, myogenesis, somitogenesis, sex determination, vasculogenesis, mesoderm formation, and neurogenesis (reviewed in Fisher and Caudy 1998a; Davis and Turner 2001). Misregulation of HES family members has been linked to developmental defects and oncogenesis. In *Drosophila,* the HES family consists of Hairy and twelve other structurally related proteins, including Deadpan and seven members of the Enhancer of split complex . All members of this repressor family possess a highly conserved bHLH domain, required for DNA binding and protein dimerization; an adjacent Orange domain, which confers specificity among family members; and a C-terminal tetrapeptide motif, WRPW, which has been shown to be necessary and sufficient for the recruitment of the corepressor Groucho.

HES proteins have been shown to bind preferentially to Class C sites (CACNNG; C-box) as homodimers in vitro (Sasai et al. 1992; Tietze et al. 1992; Oellers et al. 1994; Ohsako et al. 1994; Van Doren et al. 1994). The prevailing view is that Hairy functions as a promoter-bound repressor: an intact bHLH region is required for Hairy to bind to specific DNA sites, where it then recruits cofactors to mediate its activities. Indeed, *ac* has been shown to be a direct transcriptional target of Hairy during peripheral nervous system development (Ohsako et al. 1994; Van Doren et al. 1994; Fisher et al. 1996). However, while *ftz* was identified as a genetic target of Hairy during segmentation, there is currently no evidence for Hairy binding directly to the *ftz* promoter to regulate its transcription (despite the efforts of several labs to find such an association).

A common theme among DNA-bound transcriptional regulators is the recruitment of coactivators or corepressors to carry out their functions (reviewed in Mannervik et al. 1999; Bone and Roth 2001; Urnov et al. 2001; Jepsen and Rosenfeld 2002). Three such cofactors have been identified as Hairy-interacting proteins that are required for Hairy-mediated transcriptional repression: Groucho, *Drosophila* C-terminal binding protein (dCtBP), and *Drosophila* silent information regulator 2 (dSir2) (Paroush et al. 1994; Poortinga et al. 1998, Phippen et al. 2000, Rosenberg and Parkhurst 2002). None of these cofactors bind DNA themselves, but they are brought to the DNA through their interaction with sequence-specific DNA binding repressors, such as Hairy.

Groucho was the first cofactor shown to be required for Hairy-mediated repression, where it was shown to enhance the *hairy* mutant phenotype (Paroush et al. 1994). Groucho, as well as its mammalian homologs collectively called TLEs (TLE1–4), share a similar overall domain structure (reviewed in Parkhurst 1998; Fisher and Caudy 1998b). Groucho has been proposed to utilize a chromatin remodeling mechanism through its recruitment of Rpd3 (*Drosophila* histone deacetylase 1 homolog), but the evidence for the significance of this interaction is somewhat mixed (Chen et al. 1999; Mannervik and Levine 1999; Courey and Jia 2001).

C-terminal binding protein (CtBP) family members are an interesting new class of transcriptional coregulators that encode nicotinamide adenine dinucleotide^+^–dependent (NAD^+^-dependent) acid dehydrogenases (reviewed in Turner and Crossley 2001; Chinnadurai 2002a, 2002b; Kumar et al. 2002). CtBP proteins function as context-dependent cofactors: they act as either coactivators or corepressors of transcription, with distinct regions of the CtBP protein being required for activation or repression (Nibu et al. 1998a, 1998b; Poortinga et al. 1998; Phippen et al. 2000; Chinnadurai 2002a). The mechanism of CtBP coactivation is not known. CtBP proteins, however, have also been postulated to use a chromatin-based mechanism when functioning as a corepressor for transcription: they can bind to histone deacetylases and have been shown to modify histones (Sundqvist et al. 1998; Shi et al. 2003).

Like its yeast homolog, *dSir2* encodes NAD^+^-dependent histone deacetylase activity that is required for heterochromatic silencing (Rosenberg and Parkhurst 2002; Newman et al. 2002; reviewed in Gottschling 2000; Imai et al. 2000; Denu 2003). While yeast silent information regulator 2 (Sir2) has been thought to function as a dedicated heterochromatic silencing factor, *dSir2,* and more recently the human Sir2-related protein SIRT1, have been shown to play a role in euchromatic repression by interacting with Hairy and other HES family members (Rosenberg and Parkhurst 2002; Takata and Ishikawa 2003). *dSir2* mutants are viable (Newman et al. 2002) and exhibit a dominant genetic interaction with *hairy,* resulting in derepression of Ftz expression (Rosenberg and Parkhurst 2002), suggesting that Sir2 in higher organisms plays a role in both euchromatic repression and heterochromatic silencing.

The choice of cofactor recruited by a particular DNA-bound repressor has been proposed to help distinguish among the mechanisms of repression employed. Despite the importance of Hairy and other HES family proteins in many developmental regulatory processes, little is known about the number and kinds of target genes they regulate. Understanding the spectrum of direct targets will be essential to addressing mechanistic questions such as how or when different cofactors are recruited. To this end, we have used the chromatin profiling technique DamID to systematically identify direct Hairy transcriptional target genes and to obtain a global view of the cofactors Hairy recruits to the various loci at which it acts.

## Results

### Identification of Direct Hairy Transcriptional Targets in *Drosophila* Kc Cells Using the Chromatin Profiling Technique DamID

To identify direct transcriptional targets for Hairy in vivo, we employed a powerful new chromatin profiling technique, DamID, in which E. coli DNA adenine methyltransferase (Dam) tethered to a chromatin binding protein leads to specific methylation of DNA adjacent to the protein binding/recruitment sites (van Steensel and Henikoff 2000; van Steensel et al. 2001). We generated a functional Dam–Hairy fusion construct under the control of the heat shock promoter to use in *Drosophila* Kc cells (see Materials and Methods). Since overexpression of Dam fusion constructs leads to a high level of nonspecific methylation (van Steensel and Henikoff 2000), only low-level leaky expression from the uninduced heat shock promoter was used: the cells were not heat shocked. Genomic DNA was isolated from the Kc cells 24 h post transfection, and methylated DNA fragments were recovered on a sucrose gradient following digestion of the genomic DNA with the methylation-sensitive enzyme DpnI. These methylated fragments were labeled with the Cy5 (Dam–Hairy fusion protein) and Cy3 (Dam alone, a control for nonspecific binding/accessibility; van Steensel and Henikoff 2000) fluorochromes, then cohybridized to a *Drosophila* microarray chip containing approximately 6200 full-length *Drosophila* Gene Collection (DGC) cDNAs and ESTs (DGC Release 1; Rubin et al. 2000) representing roughly half of the fly cDNAs. Putative targets were identified based on the Cy5:Cy3 fluoresence ratio (van Steensel and Henikoff 2000; van Steensel et al. 2001). The DamID chromatin profiles were generated as previously described (van Steensel et al. 2001; Orian et al. 2003) and subjected to a series of statistical analyses to determine the statistically significant targets (see Materials and Methods; Datasets S1 and S2).

We identified 40 statistically significant putative direct Hairy transcriptional targets in Kc cells (Table 1). For just over half of these putative Hairy targets, some genetic, molecular, or functional information exists, allowing us to divide them roughly into three functional categories: those affecting morphogenesis (e.g., *egghead [egh], kayak, pointed, mae*), those affecting cell cycle or cell growth (e.g., *string (stg), ImpL2, Idgf2*), and those with unknown/unlinked functions. Unfortunately, the two previously identified Hairy targets, *ftz* and *ac,* are not present in the DGC Release 1 cDNA set used to generate our microarray chips.

**Table 1:**
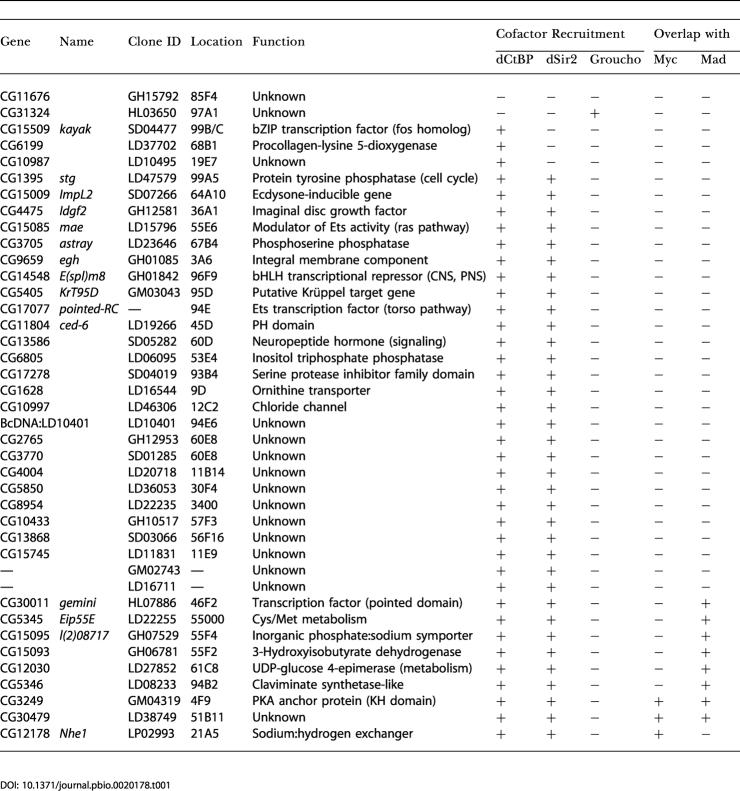
Hairy Targets Identified in Kc Cells

**Figure 1:**
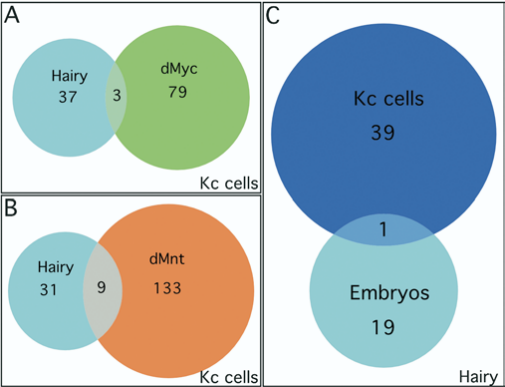
Hairy Binds to a Specific Set of Transcriptional Targets (A and B) Comparison of DamID-identified targets for Hairy with the *Drosophila* Myc and Mad/Mnt family proteins. Venn diagram comparing DamID-identified Hairy downstream targets in Kc cells compared to the transcriptional activator dMyc (A) and the transcriptional repressor dMnt (B). (C) Venn diagram comparing DamID-identified Hairy targets from Kc cells and embryos.

DamID was recently used to identify targets for the *Drosophila* Myc/Max/Mad-Mnt network of bHLH leucine zipper proteins (Orian et al. 2003), which shares many structural and functional similarities with the HES network of bHLH proteins (Gallant et al. 1996). Using the same *Drosophila* cDNA microarray chips, Orian et al. (2003) found that hundreds of binding sites are occupied by dMyc (287 targets) or dMnt (429 targets), and that their expression is modulated by dMyc in the *Drosophila* larva. Their study is consistent with a global role for Myc family proteins in modulating chromatin responsiveness of targets, and identified most of the transcriptional targets that had been found previously utilizing other approaches. As our current knowledge of direct Hairy transcriptional targets for comparison is minimal, we applied a higher stringency than Orian et al. (2003) when analyzing our Hairy DamID datasets so that we would reduce the likelihood of getting false positives. However, at this stringency we may be missing some bona fide Hairy targets. We compared the Hairy targets we identified with those identified for dMyc and dMnt using datasets analyzed at the higher statistical stringency (Figure 1). As might be expected, there was minimal overlap of Hairy targets with those identified for the transcriptional activator dMyc (three of 40 Hairy targets) (Figure 1A). There was also little overlap of Hairy targets with those identified for the transcriptional repressor dMnt (nine of 40 Hairy targets) (Figure 1B). Even when the less stringent statistics were applied to the datasets, we did not see additional overlap (data not shown). Thus, sequence-specific DNA binding factors are exhibiting binding specificity in the DamID assay, and the 40 statistically significant putative direct Hairy transcriptional targets we identified are what might be expected for a nonglobally acting sequence-specific DNA binding developmental repressor.

### Identification of Direct Hairy Transcriptional Targets in Early Embryos Using DamID

Since Hairy is part of the segmentation gene transcriptional regulatory cascade, we expected to find segmentation-related transcription factors as downstream targets of Hairy. The putative Hairy targets we identified in Kc cells do not fulfill this expectation, but rather suggest roles for Hairy in cell cycle, cell growth, and morphogenesis; these putative targets are likely targets for Hairy during its other developmental roles. This could be because of cellular context (i.e., Kc cells are thought to be embryonic neuronal stem cell in origin and may reflect Hairy's later role in neurogenesis rather than segmentation), or because only half of the *Drosophila* cDNAs are present on the chip (and the ones responding to Hairy during segmentation are not in this subset), or because the mechanism by which Hairy acts during segmentation is different than expected. To begin distinguishing among these possibilities, we used the DamID approach to identify Hairy targets in *Drosophila* embryos during segmentation.

Towards this aim, we generated functional transgenic flies carrying a UAS–Dam or UAS–Dam–Hairy fusion gene construct (see Materials and Methods). As with the Kc cells, we did not drive overexpression of these Dam fusion constructs, but rather relied on the leaky expression from the minimal promoter of the pUASp vector. Genomic DNA was harvested from 2–6-h embryos (at and just after peak Hairy expression during segmentation), then used to generate probes for the microarray chips, similar to the procedure used for the Kc cells (see Materials and Methods; Datasets S1 and S3).

We identified 20 putative direct Hairy targets from the 2–6-h embryos, which fell into four broad functional categories: transcription factors, cell cycle or cell growth, morphogenesis, and unknown/unlinked functions (Table 2). When compared to the 40 Hairy targets identified in Kc cells, we found that only one target, *egh,* overlapped between the datasets (Figure 1C). This result suggests, perhaps not surprisingly, that transcriptional targets exhibit context dependence/tissue specificity, and that the DamID approach is sensitive to developmental context/tissue specificity.

**Table 2:**
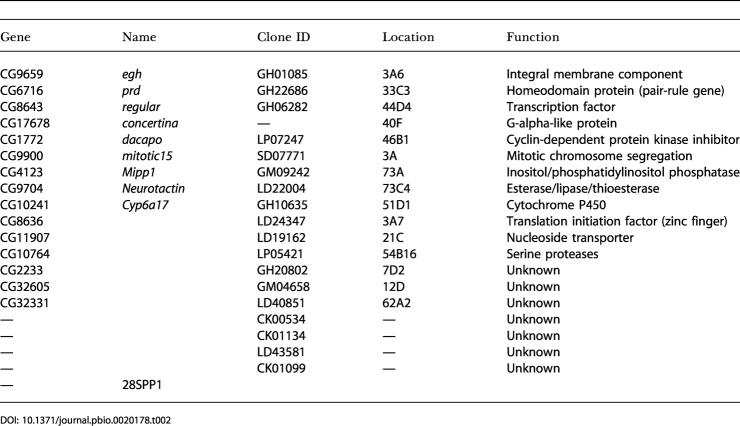
Hairy Targets Identified in Embryos (2–6 h)

Taken together, the DamID profiles for Hairy targets from Kc cells and embryos identified 59 potential new direct targets of Hairy regulation. Importantly, one of the putative Hairy targets in embryos, *paired (prd),* is a homeobox-encoding transcription factor known to function in segmentation (cf. Baumgartner and Noll 1990).

### The Expression of Potential Target Genes Depends on Hairy Regulation In Vivo

**Figure 2:**
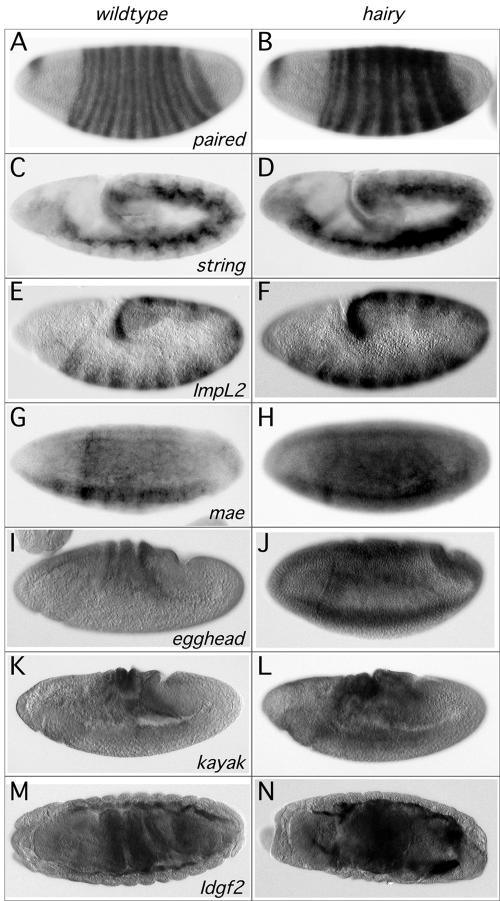
Expression of Hairy Target Genes Is Disrupted in *hairy* Mutant Embryos Whole mount in situ hybridization on wild-type (A, C, E, G, I, K, and M) or *hairy^7H^* mutant (B, D, F, H, J, L, and N) embryos with probes recognizing *prd* (A and B), *stg* (C and D), *ImpL2* (E and F), *mae* (G and H), *egh* (I and J), *kayak* (K and L), or *Idgf2* (M and N). Anterior is to the left. Dorsal is up, except in (M) and (N), which are dorsal views.

Direct Hairy targets would be expected to exhibit altered expression in a *hairy* mutant background compared to wild-type. For a subset of targets from both the Kc cell and embryo DamID experiments, we performed whole mount RNA in situ hybridization on wild-type and *hairy* mutant embryos (*hairy^7H^;* Figure 2 and data not shown). For embryo targets, we examined early embryos representing the same stages used for the DamID analysis. In keeping with our primary focus on Hairy's role in segmentation, we chose as the subset of Kc target genes to examine genes known to be expressed in the embryo (but not necessarily as early as the embryo targets), since we would not expect all of the Kc cell targets to be expressed during embryogenesis. In all cases examined, the alterations in the levels, as well as spatial and temporal patterns, of putative target gene expression were consistent with derepression in a *hairy* mutant background (Figure 2). For example, as previously described (Lehman et al. 1999), segmental expression of *stg* is altered (expanded) in a *hairy* mutant background (Figure 2C and 2D). Similarly, for *prd,* there is a failure of stripe sharpening consistent with a role for Hairy in *prd* repression and stripe maintenance (Figure 2A and 2B; Gutjahr et al. 1993).

### *hairy* Exhibits Dominant Genetic Interactions withMutants Encoding Target Genes and Affects *stg-lacZ* Reporter Expression

If Hairy is a direct regulator of a particular target gene, genetic interaction might be expected between *hairy* and a mutant corresponding to this putative target. Reduction of *hairy* dose might be expected to deregulate the expression of its target gene, resulting in increased or spatially aberrant expression of its target gene. We examined seven of the 15 Hairy targets for which mutant alleles are available for genetic interaction with *hairy* (Table 3). In all seven cases, we observed dominant genetic interactions where a reduced number of transheterozygous progeny survive (i.e., synthetic lethality). Embryos from mothers heterozygous for either *hairy* (*hairy/+*) or its target gene (i.e., *prd/+*) alone were viable. The reduction of Hairy in this target-gene-sensitized background allows inappropriate target gene regulation (i.e., target gene expression in spatial domains where it should not be expressed, with subsequent embryo lethality).

**Table 3:**
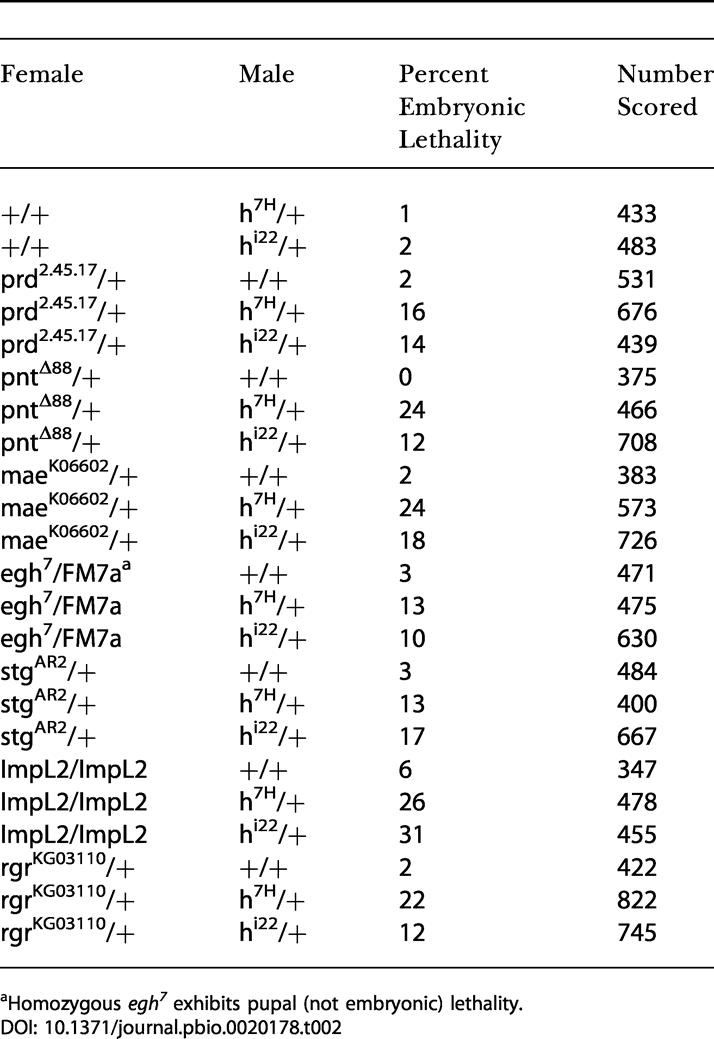
Dominant Genetic Interactions between *hairy* and Mutants Corresponding to Its Putative Downstream Targets

^a^Homozygous *egh^7^* exhibits pupal (not embryonic) lethality

**Figure 3:**
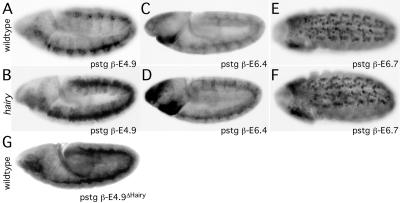
*hairy* Affects *stg-lacZ* Reporter Expression (A–F) β-galactosidase expression from the *stg-lacZ* reporter lines pstg β-E4.9 (A and B), pstg β-E6.4 (C and D), and pstg β-E6.7 (E and F) in wild-type (A, C, and E) and *hairy* mutant (B, D, and F) embryos. Note the expanded (de-repressed) lacZ expression in the *hairy* mutant background compared to wild-type for the E4.9 and E6.4 lines (compare [B] to [A] and [D] to [C], respectively). (G) β-galactosidase expression from the *stg-lacZ* reporter line pstg β-E4.9^ΔHairy^ (same as the reporter construct shown in [A], but with a Hairy binding site mutation) in a wild-type background. Note the expanded (de-repressed) lacZ expression (compare with [A]). Anterior is to the left. Dorsal is up in (A–D) and (G), whereas the ventral surface is shown in (E–F).

For one Hairy target identified in Kc cells, *stg,* a series of transgenic lines have been generated in which *lacZ* expression is driven from different promoter fragments (Lehman et al. 1999). For *stg* to be a direct transcriptional target of Hairy, we would expect Hairy to bind to the *stg* promoter. To narrow down regions of the *stg* promoter sensitive to Hairy, we examined the expression of four of these *stg-lacZ* reporter genes in *hairy* mutant and wild-type backgrounds. Sequence analysis of the promoter fragments for each of the four reporter genes revealed the presence of canonical Hairy binding sites in two of them (pstg β-E4.9 and pstg β-E6.4), but not the other two (pstg β-E2.2 and pstg β-E6.7). Consistent with the presence of Hairy binding sites, the lacZ expression from pstg β-E4.9 and pstg β-E6.4, but not from pstg β-E2.2 or pstg β-E6.7, was derepressed (expanded) in a *hairy* mutant background compared to wild-type (Figure 3; data not shown). We mutated the C-box (Hairy binding site) in the pstg β-E4.9 reporter construct (CACGCG→C**T**CGC**A**) to generate pstg β-E4.9^Δhairy^. This mutation abolishes Hairy binding in vitro (see next section and Materials and Methods). Wild-type flies carrying this pstg β-E4.9^Δhairy^ reporter exhibit the same lacZ derepression as observed for the original pstg β-E4.9 reporter when in a *hairy* mutant background, indicating that the derepression is due to Hairy binding (Figure 3G).

### Hairy Binds Directly to Target Genes

**Figure 4:**
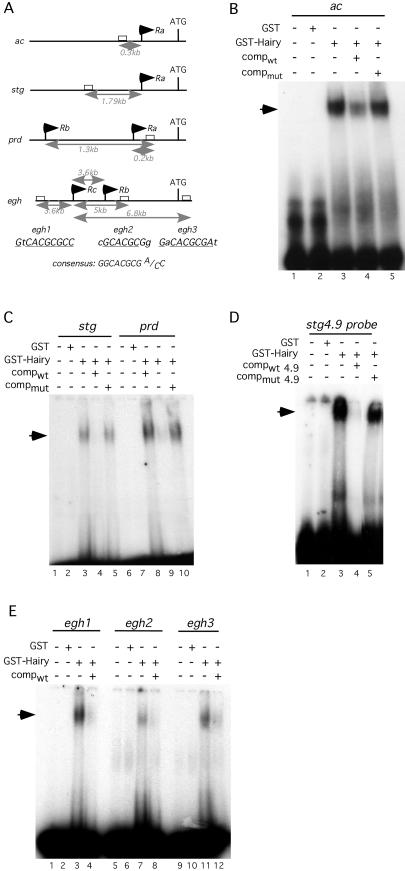
Binding of Hairy to Class C (C-Box) Sites in Putative Targets In Vitro (A) Schematic diagram (not to scale) of C-boxes within putative Hairy targets. C-boxes (Hairy binding sites) are denoted by white boxes, black arrows indicate transcription start sites (Ra, Rb, and Rc), ATG denotes the initiating methionine, and capital letters indicate bases matching with the Hairy consensus C-box. The distances in kilobases of the C-boxes from transcription start sites are noted in gray. (B) EMSA with either GST or GST–Hairy and the *ac* h/E-1 oligonucleotide. Lane 1, probe alone; lane 2, binding to probe by GST; lanes 3–5, binding to probe by GST–Hairy. In lanes 4 and 5, binding to probe by GST–Hairy was in the presence of competitor unlabeled oligos. An arrow indicates the Hairy–DNA complex; comp_wt_ and comp_mut_ indicate wild-type and mutated cold probes, respectively. (C) EMSA with either GST or GST–Hairy to the C-boxes within the *stg* and *prd* genes. Lanes 1–5, GST and GST–Hairy binding to the *stg* C-box (location: 25072658); lanes 6–10, GST and GST–Hairy binding to the *prd* C-box. (location: 12074032). Lane order and annotations are as in Figure 4B. (D) EMSA with GST–Hairy to the same C-box within the *stg* 4.9-kb genomic fragment is not competed by the presence of mutant competitor unlabeled oligo. Lane 1, probe alone; lane 2, binding to GST; lane 3, binding to probe by GST–Hairy; lanes 4 and 5, binding to probe by GST–Hairy in the presence of wild-type and mutant competitor unlabeled oligos, respectively. (E) Differential binding to C-boxes within the *egh* gene. EMSA with either GST or GST–Hairy to C-boxes within the *egh* promoter and transcribed region. Binding to three putative C-box sites is shown: egh1 (location: 2341609), egh2 (location: 2350367), and egh3 (location: 2352168). Lanes 1, 5, and 9: probe alone; lanes 2, 6, and 10: binding to probes by GST; lanes 3, 7, and 11: binding to probes with GST–Hairy. Lanes 4, 8, and 12: binding with GST–Hairy in the presence of unlabeled wild-type competitor. C-box locations and promoter information generated using Apollo (Berkeley *Drosophila* Genome Project).

Hairy has been shown to bind at Class C sequences (ggCACGCG^A^/_C_C) that contain the canonical core Hairy site (CACGCG). We searched for this consensus site within the promoter and transcribed regions of three Hairy targets: *stg, egh,* and *prd*. We identified one site in *prd,* three in *egh,* and four in the *stg* genomic region (Figure 4A). In the latter case, we focused on the site within the 4.9-kb promoter fragment, as its segmental expression was derepressed in a *hairy* mutant background (see above). We tested whether the identified sites are direct Hairy binding sites in electromobility shift assays (EMSAs), utilizing bacterially purified full-length Hairy protein and ^32^P-labeled oligos containing the appropriate Hairy binding sites (see Materials and Methods). The C-box within the *ac* promoter, a bona fide Hairy target (Ohsako et al. 1994; Van Doren et al. 1994), served as our positive control. A slow migrating complex was observed when the *ac* probe was incubated with GST–Hairy protein, but not with GST alone (Figure 4B, compare lanes 2 and 3). This binding is specific: the complex is competed by excess unlabeled wild-type *ac* oligo, but not by excess mutated *ac* oligo (Figure 4B, lanes 4 and 5, respectively). Similar assays showed direct and specific binding to the sole C-box site within the *prd* promoter, as well as to the site within the *stg* 4.9-promoter region (Figure 4C). While an oligo containing the wild-type Hairy binding site efficiently competes with Hairy binding to the *stg* 4.9-promoter region in EMSAs, an oligo encoding the mutated Hairy site used in the pstg β-E4.9^Δhairy^ reporter is unable to compete (Figure 4D). Three putative sites were identified within the *egh* promoter. Hairy binding to these sites was differential, and can be summarized as egh1 > egh3 > egh2 (Figure 4E; compare lanes 3, 7, and 11). This preferential binding may reflect sequences flanking the core C-box (CACGCG; see Figure 4A). Indeed, experiments with the related fly Enhancer of split proteins have shown that even subtle sequence changes within the core C-box or flanking sequences have dramatic consequences for the overall range of proteins that can bind in vivo (Jennings et al. 1999). We have used several bioinformatics approaches to analyze Hairy target gene promoters, to determine if there are conserved sequences flanking the core Hairy binding sites or association of the Hairy binding sites with other transcription factor binding sites as defined by the TRANSFAC database that correlate with the context dependence of Hairy binding. However, we have been unable to uncover any common features of regulation, perhaps because of the relatively small sample size of Hairy targets for these types of approaches (see Materials and Methods; data not shown).

### Hairy Binds to Specific Sites on Polytene Chromosomes

**Figure 5:**
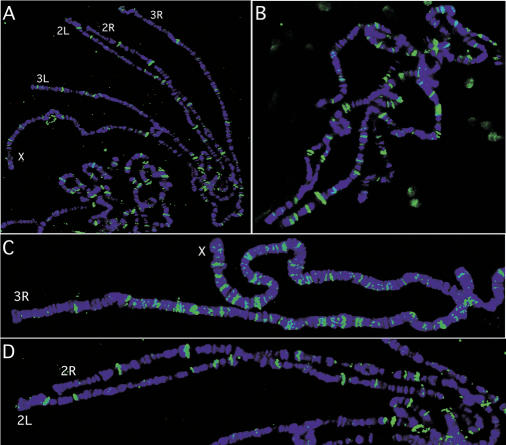
Hairy Binds to Specific Loci on Polytene Chromosomes (A and B) Hairy staining (green) on third instar larval salivary gland polytene chromosome sets counterstained with DAPI (blue) to visualize the chromosomes. (C and D) Higher magnification of chromosome arms X, 3R (C) and 2L, 2R (D).

To confirm the genomic loci associated with Hairy in vivo, we examined binding of endogenous Hairy to third instar larval salivary gland polytene chromosomes using antibodies to Hairy (Figure 5). We identified approximately 120 strongly staining sites for Hairy (Figure 5). This is likely an underestimate as some bands stain more intensely than others and likely represent more than one closely spaced binding site. Hairy binding sites are, for the most part, distributed evenly along all chromosome arms (Figures 5 and 9A).

**Figure 6:**
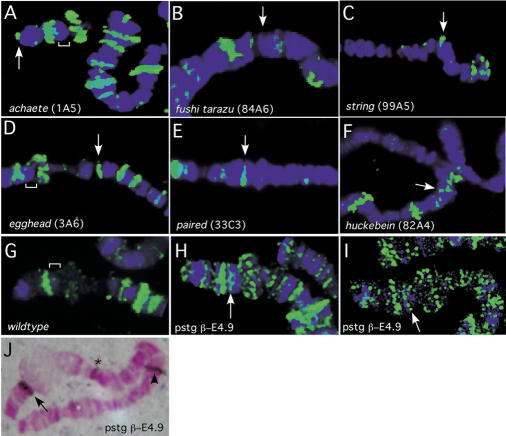
Hairy Binds to Putative Target Loci on Polytene Chromosomes (A) Hairy binds to polytene region 1A, the location of the Hairy target, *ac.* (B) Hairy is not found at 84A, the cytological location for *ftz.* (C–F) Hairy also binds to polytene region 99A, the location of *stg* (C); polytene region 3A, the location of *egh* (D); polytene region 33C, the location of *prd* (E); and polytene region 82A, the location of *hkb* (F). (G–I) Hairy is recruited to the insertion site for the pstg βE-4.9 reporter construct (arrow in [H] and [I]). Compare to the equivalent region of wild-type X chromosomes marked by brackets in (A), (D), and (G). (J) In situ hybridization to polytene chromosomes from pstg βE-4.9 larvae showing that this line has two insertions on the X chromosome at 1F and 6C. The probe also recognizes sequences to the endogenous *white* locus (asterisk).

Since there are a relatively small number of Hairy binding sites on the polytene chromosomes, the location of the bands can be determined cytologically with relatively high resolution. While we have not been able to unambiguously assign all of the approximately 120 binding sites cytologically, we examined whether Hairy staining corresponds to the targets identified in the Kc cells and embryo DamID experiments. There are 39 out of 40 Kc cell and 20 out of 20 embryo targets that map cytologically to regions that correspond to Hairy binding sites (e.g., Figure 6A–6F). Thus, while tissue or developmental specificity appear to be lost, polytene chromosomes provide a reliable indicator for Hairy DNA binding targets. Note the presence of Hairy binding at the tip of the X chromosome, the cytological location of the direct Hairy transcriptional target *ac* (Figure 6A). Interestingly, we were unable to detect Hairy binding at position 84A, the cytological location for *ftz* (Figure 6B). Hairy binding was also detected at the cytological location for *stg* (Figure 6C) and *egh* (Figure 6D), as well as at 33C, the cytological location of *prd* (Figure 6E). Recent work established a role for Hairy in regulating salivary gland tube morphology that genetically depends, in part, on repression of *huckebein (hkb),* a zinc-finger-encoding transcription factor (Myat and Andrew 2002). It is not yet known if Hairy's repression of *hkb* is direct or not. *hkb* is not in the DGC Release1 cDNA set used to generate our microarray chips, but we do find that one of the strong Hairy binding sites maps to 82A on polytene chromosomes, the cytological location of *hkb* (see Figure 6F). Consistent with our identification of *stg* as a Hairy target, derepression of C-box-containing *stg-lacZ* reporter lines, and gel shift assays, we detect a new band of Hairy staining in chromosomes from larvae carrying the *stg-lacZ* (pstg β-E4.9) reporter at cytological location 1F, the transgene insertion site (Figure 6G–6J; see Materials and Methods).

### Identification of Targets for Recruitment of the Transcriptional Cofactors Groucho, dCtBP, and dSir2

**Figure 7:**
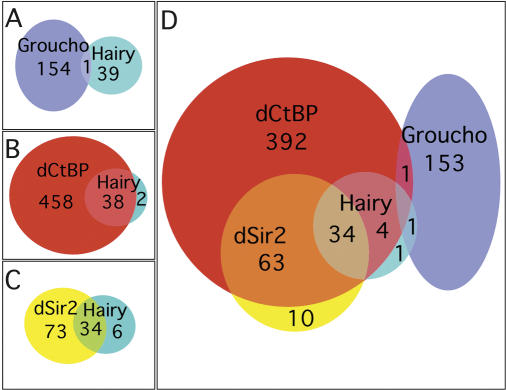
Hairy Overlaps with Cofactors Differentially (A–C) Venn diagram showing the overlap between Hairy targets and those loci also binding to the cofactors Groucho (A), dCtBP (B), and dSir2 (C). (D) Venn diagram showing combined overlaps of Hairy with its three known cofactors.

As with other sequence-specific DNA binding transcription factors, Hairy recruits cofactors to carry out its functions. One of the major questions in the field concerns how and when particular cofactors are recruited. It has been technically challenging to address this question with current methods such as ChIP assays, since cofactor association may be transient, unstable, or far removed from the DNA binding protein. Utilizing expression-based microarray analysis is also not easy, because of the difficulty in sorting direct from indirect interactions with such widely recruited cofactors. To circumvent these technical issues and as a first step towards understanding the rules governing Hairy cofactor recruitment, we used the DamID approach to determine if the three known Hairy cofactors, Groucho, dCtBP, and dSir2, are recruited to all or a subset of Hairy targets. We generated Dam fusions to Groucho and dCtBP (see Materials and Methods). The Dam–dSir2 fusion construct was described previously (van Steensel et al. 2001). While none of these cofactors binds DNA on its own, they are recruited to the DNA through their interaction with sequence-specific DNA binding proteins such as Hairy. Using the same procedure and statistical analyses used for the identification of Hairy targets in Kc cells (see Material and Methods; Datasets S1 and S4–S6), we identified 155 loci that recruit Groucho, 496 loci that recruit dCtBP, and 107 loci that recruit dSir2 in Kc cells (Figure 7; Datasets S7–S9). Comparison for overlap between these cofactor datasets and that of Hairy from Kc cells showed that, surprisingly, only one of the putative Hairy targets we identified overlaps with Groucho recruitment (Figure 7A and 7D). The majority of Hairy targets, however, overlap with dCtBP (38/40; Figure 7B and 7D), and most of these also overlap with dSir2 (34/40; Figure 7C and 7D). At present, we cannot rule out the possibility that a protein unrelated to Hairy is recruiting these cofactors to a given putative Hairy target. Interestingly, dCtBP and dSir2 appear to colocalize at loci outside the subset of putative Hairy targets (90% of dSir2 targets overlap with those of dCtBP; Figure 7D).

### Hairy Target Gene Expression Depends on Hairy Cofactor Regulation In Vivo

**Figure 8:**
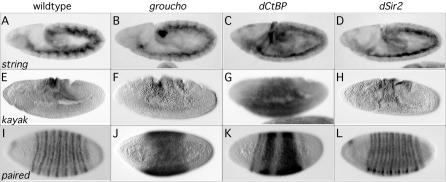
Hairy Target Gene Expression Is Disrupted in the Mutant Background of the Cofactors Associated with a Particular Target Whole mount in situ hybridization on wild-type (A, E, and I), *groucho* germline clone (B, F, and J), *dCtBP* germline clone (C, G, and K), and *dSir2* mutant (D, H, and L) embryos with probes recognizing *stg* (A–D), *kayak* (E–H), or *prd* (I–L). Anterior is to the left. Dorsal is up.

If particular Hairy targets require specific cofactors to be appropriately regulated, we would expect their expression to be altered (deregulated) in a cofactor mutant background. We performed RNA in situ hybridization for two Hairy Kc cell targets that differentially recruit Groucho, dCtBP, and dSir2. We chose to examine the expression of two *hairy* targets that are expressed relatively early in the embryo since these cofactors are used in a number of developmental systems and exhibit severe morphological phenotypes when their activity is removed maternally (cf. Phippen et al. 2000). Consistent with a requirement for dCtBP and dSir2, *stg* expression is derepressed in *dCtBP* and *dSir2,* but not *groucho* mutant backgrounds (Figure 8A–8D). Similarly, consistent with a requirement for dCtBP alone, *kayak* expression is expanded in *dCtBP,* but not in *groucho* or *dSir2* mutant backgrounds (Figure 8E–8H). While we cannot extrapolate the cofactor recruitment requirements from Kc cells to embryos, we used in situ hybridization as a prediction for cofactor recruitment for the embryo target, *prd*. We examined the expression of *prd* in cofactor mutant backgrounds and found that *prd* expression is altered in *groucho* and *dCtBP,* but not *dSir2,* mutant backgrounds (Figure 8I–8L), suggesting that *prd* may represent a minority of Hairy targets that could recruit both Groucho and dCtBP. Consistent with this finding, we find both Groucho and dCtBP staining on polytene chromosomes at the cytological location for *prd* (data not shown).

### The Transcriptional Cofactors Groucho, dCtBP, and dSir2 Are Recruited to Specific Sites on Polytene Chromosomes

**Figure 9:**
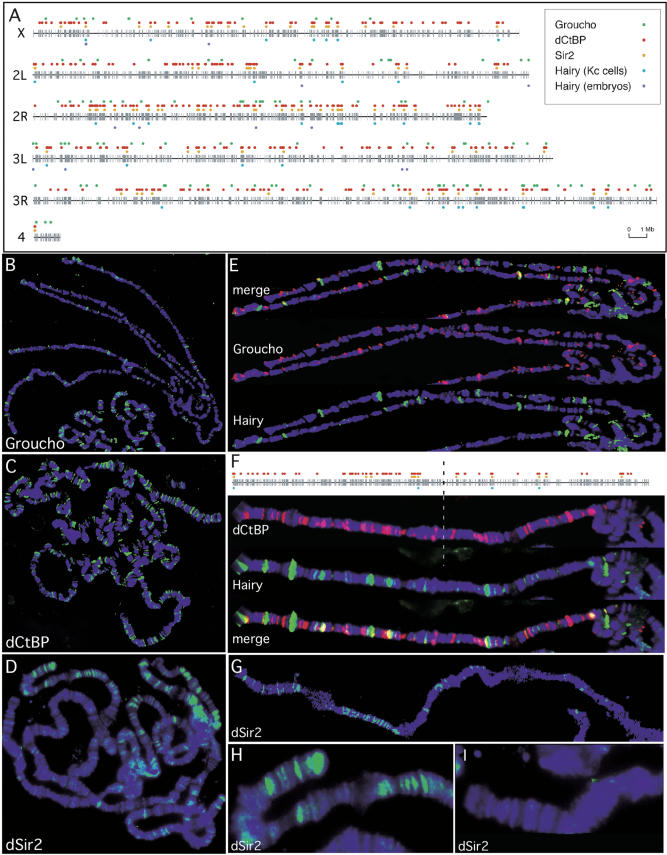
Hairy Shows Context-Dependent Association with Its Cofactors (A) Sites of Hairy binding and Hairy cofactor recruitment based on DamID. The gray lines depict the relative position on the chromosomes of the approximately 6200 cDNAs on the microarray chip. The blue dots below the line represent Hairy binding sites while the green (Groucho), red (dCtBP), and yellow (dSir2) dots represent the positions of cofactor recruitment. (B–D) Cofactor recruitment visualized on third instar larval salivary gland chromosomes. Polytene chromosome sets stained (green) with antibodies to Groucho (B), dCtBP (C), and dSir2 (D). All chromosomes were counterstained with DAPI (blue) to visualize the DNA. (E) Higher magnification view of chromosome arms 2L and 2R costained with Groucho (red) and Hairy (green), and the merged image. (F) Higher magnification view of chromosome arm 2L costained with dCtBP (red) and Hairy (green), and the merged image, compared to the predicted DamID map. Note that both the DamID projected map and polytene chromosomes have more dCtBP recruitment sites to the left of the dashed line than to the right of the dashed line. (G) Chromosome arm 3R stained with dSir2 (green), highlighting regional specificity of dSir2 recruitment. (H and I) Higher magnification view of the distal ends of chromosome arms 2R (H) and 3L (I) from (D), stained with dSir2 (green), showing regional specificity and lack of dSir2 recruitment, respectively.

When DamID data for the three Hairy cofactors and Hairy itself are graphically projected onto chromosomes, several interesting features come to light (Figure 9A). For example, while Groucho and dCtBP are distributed along all the chromosomes, dSir2 shows region- and chromosome-specific binding (e.g., there are more dSir2 sites on Chromosome 2R than on Chromosome 3L). To confirm loci associated with recruitment of the different cofactors in vivo, we examined the localization of endogenous Groucho, dCtBP, and dSir2 on wild-type third instar larval salivary gland polytene chromosomes using antibodies to Groucho, dCtBP, and dSir2, respectively (Figure 9B–9D). Consistent with the relative numbers of targets identified for each of the cofactors by the DamID approach, we find many more sites for dCtBP than either Groucho or dSir2. Also consistent with our DamID findings, Groucho overlaps with Hairy at only a small number of the Hairy binding sites (Figure 9E), whereas dCtBP overlaps with the majority of Hairy binding sites (Figure 9F). Differences in distribution for the cofactors observed by DamID are reflected on the polytene staining patterns. For example, our DamID data suggest that the distal portion of Chromosome 2L has more sites for dCtBP than the proximal half of the chromosome. This observation is reflected in dCtBP recruitment on the polytene chromosomes as well (Figure 9F). Likewise, as predicted from the DamID data, dSir2 staining on the polytene chromosomes exhibits region-specific association in which some chromosomes and chromosomal regions exhibit a high degree of staining, while other whole chromosomes exhibit very little staining (Figure 9D and 9G–9I; Rosenberg and Parkhurst 2002).

## Discussion

We have known for almost two decades that Hairy plays a pivotal role in the segmentation hierarchy, as well as other developmental processes, but the details of Hairy action have not been easy to tease apart. An important step in understanding the molecular mechanisms surrounding Hairy-mediated repression was made with the identification of Groucho as a Hairy binding protein (Paroush et al. 1994). One of the key remaining questions regarding the mechanism(s) of repression employed by Hairy concerns the identities of its direct transcriptional targets. We have employed a novel chromatin profiling approach, DamID, to effectively identify a total of 59 potentially direct Hairy targets from 2–6-h embryos and Kc cells. As expected of direct targets, these genes show Hairy-dependent expression, are detected as Hairy binding sites in vivo on polytene chromosomes, and have consensus C-box-containing sequences that are directly bound by Hairy in vitro. While the DamID approach had previously been used only in Kc cells, we found that this technique is also powerful when utilizing transgenic embryos that carry fusions of the protein of interest to the Dam methylase. As target genes are likely context dependent, the use of embryos makes it possible to choose the precise time or place of development to be examined, as well as allowing the analysis to take place in an organismal context.

The 59 putative Hairy targets we identified in the embryo and Kc cell DamID experiments correspond to bands of Hairy immunostaining on polytene chromosomes, suggesting that the polytene chromosome staining faithfully represents Hairy binding. Polytene chromosomes are functionally similar in transcriptional activity and display factor/cofactor binding properties similar to chromatin of diploid interphase cells, despite their DNA endoreplication (Hill et al. 1987; Andrew and Scott 1994; Hill and Mott 2000; Pile and Wassarman 2000, 2002). Since the microarray chips we used contain roughly half of *Drosophila* cDNAs, we estimate the actual number of Hairy targets to be approximately twice that number (i.e., 118 targets). This predicted number of Hairy targets is close to the approximately 120 strongly staining sites we observe on polytene chromosomes. Of the 59 putative Hairy targets we identified in both the Kc cell and embryo DamID experiments, 58 correspond to bands of Hairy staining on the polytene chromosomes, suggesting that polytene chromosome staining is representing Hairy binding sites without regard to tissue specificity. It is not yet clear what is limiting Hairy accessibility in different tissues or why Hairy's access does not appear to be limited in salivary glands. It may be that polytene chromosome organization necessitates a looser chromatin structure or that the large number of factors that seem to be endogenously expressed in salivary glands affects accessibility. Ultimately, additional confirmation of the DamID and polytene staining correspondence will require microarray tiling chips containing overlapping genomic DNA fragments; however, such genomic DNA tiling chips are currently unavailable.

Van Steensel and Henikoff (2000) showed that DNA methylation by tethered Dam spreads up to a few kilobases from the point where it is brought to the DNA. We were concerned in the beginning that we might miss Hairy targets if the DNA fragments of 2.5 kb or less that we recovered for probes were far away from the start of the transcribed region, especially since the *Drosophila* microarray chip we used was generated using full-length cDNAs. Indeed, as Hairy has been described as a long-range repressor (Barolo and Levine 1997), it is likely to bind at a distance from the transcription start site. However, the targets we identified by DamID in both Kc cells and in embryos correspond closely to the Hairy staining pattern on polytene chromosomes. As is the case for Hairy, the distribution of DamID-identified loci that recruit the long-range repression-mediating Groucho corepressor (Zhang and Levine 1999) corresponds well with the distribution of Groucho binding sites on polytene chromosomes. Our results suggest that there is a higher-order structure to the promoter that is allowing factors that bind far upstream of the transcription start site to have physical access to the transcribed region (i.e., DNA looping; reviewed in Ogata et al. 2003) or that Hairy does not bind as far away from the transcription start site as it has been proposed to do.

### Hairy Targets

Hairy is needed at multiple times during development, where it has primarily been associated with the regulation of cell fate decisions. During embryonic segmentation, *ftz* has long been thought to be a direct Hairy target. However, the order of appearance of *ftz* stripes is not inversely correlated with those of Hairy, as would be expected if *ftz* stripes are generated by Hairy repression (Yu and Pick 1995). While we were unable to assess *ftz* as a direct Hairy target using DamID, we did not find evidence for *ftz* being a direct Hairy target based on the association of Hairy with polytene chromosomes. Indeed, the evidence suggesting that *ftz* is a direct target of Hairy is based on timing, i.e., that there is not enough time for another factor to be involved (cf. Ish-Horowicz and Pinchin 1987). As the half-life of the pair-rule gene products is very short (less than 5 min; Edgar et al. 1986), it is possible that additional factors could be acting and that the interaction between Hairy and *ftz* is indirect.

Interestingly, one of the Hairy targets we identified in embryos is the homeobox-containing transcriptional regulator, *prd*. Pair-rule genes have been split into two groups: primary pair-rule genes mediate the transition from nonperiodic to reiterated patterns via positional cues received directly from the gap genes, whereas secondary pair-rule genes take their patterning cues from the primary pair-rule genes and in turn regulate the segment polarity and homeotic gene expression. The transcriptional regulator *prd* was originally categorized as a secondary pair-rule gene since its expression is affected by mutations in all other known pair-rule genes. However, *prd* stripes were subsequently shown to require gap gene products for their establishment, and the *prd* locus has the modular promoter structure associated with primary pair-rule genes (Baumgartner and Noll 1990; Gutjahr et al. 1993). Thus, *prd* has properties of both primary and secondary pair-rule genes and is a good candidate to directly mediate Hairy's effects on segmentation. We found that Hairy can specifically bind to C-box sequences in the *prd* promoter and interacts genetically with *prd*. Further experiments will be required to determine if Paired in turn binds to the *ftz* promoter, such that the order of regulation would be Hairy > *prd* > *ftz*.

In addition to identifying potential targets for Hairy in segmentation, we identified targets that implicate Hairy in other processes including cell cycle, cell growth, and morphogenesis. The group of targets implicating Hairy in the regulation of morphogenesis includes: *concertina,* a G-alpha protein involved in regulating cell shape changes during gastrulation (Parks and Wieschaus 1991); *kayak,* the *Drosophila* Fos homolog involved in morphogenetic processes such as follicle cell migration, dorsal closure, and wound healing (Riesgo-Escovar and Hafen 1997; Dequier et al. 2001; Dobens et al. 2001; Ramet et al. 2002); *pointed* and *mae,* both of which function in the *ras* signaling pathway to control aspects of epithelial morphogenesis (cf. Beitel and Krasnow 2000; Baker et al. 2001; James et al. 2002); *egh,* a novel, putative secreted or transmembrane protein proposed to play a role in epithelial morphogenesis (Goode et al. 1996); and *Mipp1,* a phosphatase required for proper tracheal development (Ebner et al. 2002).

Hairy has been thought to be involved mostly in the regulation of cell fate decisions. However, mosaic experiments in the eye imaginal disc have suggested that Hairy may also play a role in the regulation of cell cycle or cell growth (Brown et al. 1995). Consistent with this, another group of Hairy targets implicates Hairy in the regulation of cell cycle or cell growth; this group includes *stg,* the *Drosophila* Cdc25 homolog (cf. Lehman et al. 1999); *dacapo,* a cyclin-dependent kinase inhibitor related to mammalian p27^kip1^/p21^waf1^ (Lane et al. 1996; Meyer et al. 2002); *IDGF2,* a member of a newly identified family of growth-promoting glycoproteins (Kawamura et al. 1999); and *ImpL2,* a steroid-responsive gene of the secreted immunoglobulin superfamily that functions as a negative regulator of insulin signaling (Garbe et al. 1993; Andersen et al. 2000; Montagne et al. 2001; Tapon et al. 2001; Johnston and Gallant 2002). Consistent with a role for Hairy in growth signaling, mammalian HES family proteins have been linked to insulin signaling (Yamada et al. 2003).

Since cells that are dividing or proliferating cannot simultaneously undergo the cell shape changes and cell migrations required for morphogenetic movements, Hairy may be required to transiently pause the cell cycle in a spatially and temporally defined manner, thereby allowing the cell fate decisions regulated by the transcription cascade to be completed. As Hairy is itself spatially and temporally expressed, Hairy must be only one of several genes necessary to orchestrate these processes. While much progress has been made in understanding the regulatory networks governing pattern formation, cell proliferation, and morphogenesis, and while it is clear that they must be integrated, the details surrounding their coordination have not yet been elucidated. Thus, the putative Hairy targets we identified are consistent with known processes involving Hairy and suggest that in addition to regulating pattern formation, Hairy plays a role in transiently repressing other events, perhaps in order to coordinate cell cycle events with the segmentation cascade. Further experiments will be needed to determine how these different roles for Hairy fit together.

### Cofactor Recruitment

Corepressor recruitment is an important aspect of transcriptional repression (reviewed in Mannervik et al. 1999; Bone and Roth 2001; Mannervik 2001; Urnov et al. 2001; Jepsen and Rosenfeld 2002). While the sequence-specific DNA-bound repressors contribute to target specificity, the corepressors are thought to help distinguish among particular repression mechanisms to be used via alteration of their recruitment or function. For example, the *Drosophila* developmental factors Dorsal and T-cell factor (TCF) have been shown to function as either positive or negative regulators of transcription depending on promoter context and cofactor recruitment (Dubnicoff et al. 1997; Cavallo et al. 1998). As each of Hairy's cofactors appears to act differently with Hairy, thereby conferring different developmental consequences, we used the DamID approach, along with polytene chromosome staining, to get our first look at the patterns of Hairy's cofactor recruitment.

The numbers of loci that recruit Groucho, dCtBP, and dSir2 cofactors are consistent with the breadth of interaction they have been shown to exhibit. We identified by DamID profiling 155 loci that recruit Groucho and, as expected, found roughly twice as many sites on polytene chromosomes. Groucho was one of the first corepressors identified and shown to affect a variety of different developmental processes, suggesting that it is a widely used corepressor (Parkhurst 1998; Chen and Courey 2000). In addition to its interaction with Hairy, Groucho was subsequently shown to mediate repression through several other classes of DNA-binding transcriptional regulators including Engrailed, Dorsal, T-cell factor, and Runt (Aronson et al. 1997; Dubnicoff et al. 1997; Jiménez et al. 1997; Cavallo et al. 1998; Roose et al. 1998).

Although Groucho was the first Hairy cofactor identified (Paroush et al. 1994) and its interaction site is often described as Hairy's “major” repression motif (Mannervik 2001), we find that it is associated with only a minority of Hairy targets in Kc cells. Groucho's dominance as a cofactor during segmentation may reflect a preference for Groucho in the reporter assays used previously to assess corepressor activity, or it may be more heavily recruited to Hairy's targets during segmentation. In the future it will be interesting to determine the loci that recruit Groucho in early embryos and, as Groucho binds a number of other repressors, which, if any, of these factors recruits Groucho as its major cofactor.

CtBP was identified more recently, first on the basis of its binding to the C-terminal region of E1A, and in *Drosophila* by its association with the developmental repressors Hairy and Knirps (reviewed in Turner and Crossley 2001; Chinnadurai 2002a). CtBP is an integral component in a variety of multiprotein transcriptional complexes. It has been shown to function as a context-dependent cofactor, having both positive and negative effects on transcriptional repression depending upon the repressor to which it is recruited. More than 40 different repressors have been shown to recruit CtBP. Consistent with this wide recruitment of CtBP, we identified 496 loci that recruit dCtBP by DamID profiling and roughly twice that many sites on polytene chromosomes. A recently reported global protein–protein interaction study showed that the binding partners for Groucho and dCtBP are largely nonoverlapping (Giot et al. 2003). This, along with the near exclusivity of Groucho and dCtBP binding as assayed by DamID and polytene chromosome staining, makes it unlikely that both cofactors work together as a general rule and strengthens the possibility that the binding of each of these factors assembles different protein complexes that are, for the most part, mutually exclusive.

dSir2 was only very recently identified as a corepressor for Hairy and other HES family members (Rosenberg and Parkhurst 2002; Takata and Ishikawa 2003). We identified 107 loci that recruit dSir2 by DamID profiling and roughly twice that many sites on polytene chromosomes. Surprisingly, the distribution of loci recruiting dSir2 identified by DamID profiling, as well as dSir2′s staining on polytene chromosomes, shows regional binding specificity (see Figure 9D and 9G). This binding specificity may be a reflection of the different nuclear compartments that these regions of the chromosomes find themselves in (cf. Francastel et al. 2000; Leitch 2000). Sir2 has been described mostly as a protein involved in heterochromatic silencing rather than in euchromatic repression. The number of dSir2 euchromatic sites we observe is similar to that of Groucho, suggesting that euchromatic repressors (in addition to HES family members) are likely to recruit Sir2. Consistent with this, a recent report has described a role for mammalian Sir2 in repressing the muscle cell differentiation program (Fulco et al. 2003). The region-specific binding of dSir2 might reflect a difference in the types of factors it can associate with, or the association of dSir2 with particular chromosomal regions or nuclear domains (cf. Spellman and Rubin 2002).

Interestingly, dCtBP and dSir2 recruitment are largely overlapping, and this association continues outside of those loci where Hairy binds: 90% of dSir2-recruiting loci also recruit dCtBP. dCtBP and dSir2 are unique among transcriptional coregulators in that they both encode NAD^+^-dependent enzymatic activities. As NAD and NADH levels within the cell exist in closely regulated equilibrium (for review see Dang et al. 1997; Ziegler 2000), it is possible that dCtBP and dSir2 function as NAD/NADH redox sensors (cf. Denu 2003; Fjeld et al. 2003). In this way, the cell could use coenzyme metabolites to coordinate the transcriptional activity of differentiation-specific genes with the cellular redox state.

The success of the combination of DamID profiling and polytene chromosome staining results provides a global systematic way in which to address a number of mechanistic questions concerning the rules governing cofactor recruitment. For example, it will be possible to address whether target gene location or promoter structure determines the accessibility of cofactors to specifically bound repressors or whether, conversely, the association of repressors with cofactors influences target gene choice by altering DNA binding specificity. We now have a number of direct Hairy targets and in vivo assay systems to use in future experiments addressing questions surrounding Hairy's biological functions and the precise molecular mechanisms it employs to carry out its functions.

## Materials and Methods

### 

#### DamID.

To generate Dam–Hairy or Dam–dCtBP, a full-length *hairy* or *dCtBP* cDNA fragment was generated by standard PCR using primers containing a BglII 5′ site and a XbaI 3′ site, cut with BglII and XbaI, and subcloned into the BglII and XbaI sites of pNMycDam plasmid, as described previously (van Steensel and Henikoff 2000). To generate Dam–Groucho, a full-length *groucho* cDNA fragment (minus the stop codon) was generated by standard PCR using primers containing a BamHI 5′ site and a NotI 3′ site, cut with BamHI and NotI, and subcloned into the BglII and NotI sites of pCMycDam plasmid, as described previously (van Steensel and Henikoff 2000). Dam–dSir2 was described previously (van Steensel et al. 2001). All four of these constructs are expressed in Kc167 cells (data not shown). Kc cell culture and transfections were performed as described previously (Henikoff et al. 2000). The Kc cells were harvested 24 h posttransfection, then genomic DNA was isolated and processed for microarray hybridizations as described previously (van Steensel et al. 2001).

The UAS–Dam and UAS–Dam–Hairy expression constructs were made by first amplifying the Dam or Dam–Hairy open reading frames by PCR from the appropriate fusion construct described above, then cloning them into the pUASp vector (Rørth 1998) as 5′KpnI-3′XbaI fragments. The resulting UAS–Dam and UAS–Dam–Hairy plasmids (500 μg/ml) were injected along with the pTURBO helper plasmid (100 μg/ml) (Mullins et al. 1989) into isogenic w^1118^ flies as described by Spradling (1986). Transgenics were scored by eye color, and the insertions were mapped and balanced using standard genetic methods. These chimeric genes are properly expressed when induced with various Gal4 driver lines (e.g., Engrailed–Gal4; Brand and Perrimon 1993; data not shown). The Dam–Hairy fusion protein is functional because presence of the UAS–Dam–Hairy transgene, but not the UAS–Dam transgene, partially rescues the segmentation phenotype of *hairy* mutant embryos when induced with an actin–Gal4 driver (rescue is similar to UAS–Hairy; data not shown). As in Kc cells, induced expression of these Dam fusion constructs leads to high levels of nonspecific methylation. Therefore we utilized low-level leaky expression from the minimal promoter of the pUASp vector for these experiments. 2–6-h embryos were collected and dechorionated with 100% bleach. Approximately 500 μl of embryos were crushed in 1 ml of lysis buffer (100 mM Tris [pH 9.0], 100 mM NaCl, 100 mM EDTA, and 5% sucrose). SDS (to 0.5%) and proteinase K (to 100 μg/ml) were added immediately after homogenization, followed by incubation at 55 °C for 2 h. SDS was increased to 1.5%, followed by incubation for an additional 2–3 h. The genomic DNA was isolated and processed for microarray hybridizations essentially as described previously (van Steensel et al. 2001).

*Drosophila* microarray chips were produced in house (Genomics Shared Resource; Fred Hutchinson Cancer Research Center, Seattle, Washington, United States) for the Northwest Fly Consortium and contain approximately 6200 full-length DGC cDNAs (DGC Release 1; Rubin et al. 2000), as well as approximately 300 clones added by members of the Consortium.

Arrays were scanned using a GenePix 4000 scanner (Axon Instruments, Union City, California, United States), and image analysis was performed using GenePix Pro 3.0. For each array, spot intensity signals were filtered and removed if the values did not exceed 3 standard deviations above the background signal in at least one channel or if the spot was flagged as questionable by the GenePix Pro software. For each spot, background-corrected ratios were natural log transformed and a median-centered normalization strategy was applied across each array. Dam–protein and Dam transfections were independently replicated three times, and the subsequent array comparisons (i.e., Dam–protein/Dam) were analyzed using CyberT (Baldi and Long 2001), a Bayesian t-statistic derived for microarray analysis (http://genomics.biochem.uci.edu/genex/cybert/). We employed the default window size of 101 and used a confidence value of ten in our CyberT analysis. The null hypothesis was rejected and a spot ratio was called significantly changed if *p*_Bon_ ≤ 0.05, where *p*_Bon_ is the Bayesian *p*-value adjusted for multiple hypothesis tests using the conservative Bonferroni correction. Based on prior “self versus self” DamID comparisons, we empirically determined a lower-bound ln(ratio) threshold = |0.405| as an additional significance criterion to discriminate spot intensity signals from the inherent noise in the hybridization process. For each protein analyzed, a fluor-reversed array comparison was performed and used to screen all significant calls for fluor-specific artifacts. For our analyses, we treated the small subset of replicated spots on the array independently. For those cases, both spots were required to be called significant. Reported ratio values were retransformed to log_2_ as a matter of convention. The complete raw and processed datasets can be accessed at http://www.fhcrc.org/labs/parkhurst/supplementary-data/.

#### Flies and genetics.

Flies were cultured and crossed on yeast-cornmeal-molasses-malt extract medium at 25 °C. The alleles used in this study were the following: *h^7H^ rucuca*/ TM3, *h^12C^ st e*/ TM3, Df(3 l)*h^i22^ Ki roe p^p^*/TM3, and *prd^2.45.17^*/CyO (D. Ish-Horowicz); FRT82B- P{ry+t7.2=PZ}*CtBP^03463^ ry^506^*/TM3 (N. Perrimon); FRT 82B- *gro^E47^*/TM3 (Phippen et al. 2000); *dSir2^5.26^*/SM6 and *dSir2^4.5^*/SM6 (Newman et al. 2002); FRT82B-*ovo^D1^*/TM3, *y w* hs-FLP22, TM3/CxD, *egh^7^*/FM7a (#3902), *ImpL2^KG02223^* (#14083), *mae^k06602^*/CyO (#10633), *pnt^Δ88^*/TM3 (#861), and *rgr^KG03110^* (#13770) (Bloomington *Drosophila* Stock Center, Indiana University, Bloomington, Indiana, United States). Details of these strains are found on FlyBase (http://flybase.bio.indiana.edu:82/). *stg^AR2^* and the *stg-lacZ* reporter lines (pstg β-E2.2, pstg β-E4.9, pstg β-E6.4, pstg β-E6.7) were described previously (Lehman et al. 1999). The genomic locations of the Hairy binding sites in pstg β-E4.9 and pstg β-E6.4 are 25072653 and 25080219, respectively. Germline clones for *dCtBP* and *groucho* were generated as previously described (Poortinga et al. 1998, Phippen et al. 2000). The pstg-βE4.9^Δhairy^ transgenic flies were generated by injecting vector (500 μg/ml) along with the pTURBO helper plasmid (100 μg/ml) (Mullins et al. 1989) into isogenic w^1118^ flies as described by Spradling (1986). Transgenics were scored by eye color, and the insertions were mapped and balanced using standard genetic methods.

#### Embryo analysis.

Larval cuticle preparations were prepared and analyzed as described by Wieschaus and Nüsslein-Volhard (1986). Immunohistochemical detection of proteins in embryos was performed as described previously (Parkhurst et al. 1990) using Alkaline Phosphatase–coupled secondary antibodies (Jackson Laboratory, Bar Harbor, Maine, United States) visualized with Substrate Kit II reagents (Vector Laboratories, Burlingame, California, United States). Antisera used were as follows: antiMyc (9e10, 1:100 dilution; Santa Cruz Biotechnology, Santa Cruz, California, United States).

Immunohistochemical whole mount RNA in situ hybridization was performed according to the protocol of Tautz and Pfeifle (1989). Digoxygenin-substituted probes were obtained by PCR amplification with primers to the vector just 3′ of the cDNA insert.

#### EMSA.

EMSA was carried out using either bacterially expressed GST or GST–Hairy ( full-length) proteins, similar to the procedure described by Van Doren et al. (1994) and Rosenberg and Parkhurst (2002). Briefly, 40 fmol of ^32^P-end-labeled probe of each oligo was incubated with either GST– or GST–Hairy–purified proteins (200 ng each), in a 25-μl reaction supplemented with binding buffer (5% glycerol, 20 mM HEPES [pH 7.6], 50 mM KCl, 1 mM EDTA, 1 mM DTT, and 10 ng/μl poly dI-dC) at room temperature. Where indicated, the binding was preformed in the presence of 15-fold excess of unlabeled wild-type or mutated *ac* competitor oligos. Following incubation, the complexes were resolved using 0.5% TBE-PAGE gels and visualized by autoradiograms. The following oligos were used (forward primers are shown): *ac* 5′-TAAACCGGTTGGCAGCCGGCACGCGACAGGGCCAGGTTTT-3′; *egh* egh1 5′-TGCGCGTCACGCGCCGTTC-3′, egh2 5′-TCATTCGCACGCGGAATCT-3′, and egh 3 5′-GCCGGACACGCGATGATGG-3′; mutated *ac* oligo 5′-TAAACCGGTTGGCAGCCGG**G**ACGCGACAGGGCCAGGTTTT-3′; mutated *stg* oligo 5′-TCTACCACACACAAACAC**T**CGC**A**CGCGAAAACTGGG -3′; *prd* 5′-AAGTGACACGCGCTCCGCT-3′; and *stg* 5′-AAACACACGCGCGCGAAAA-3′.

#### Hairy binding site bioinformatics analysis.

Several bioinformatics approaches were employed to analyze Hairy target gene promoters. In particular, *Drosophila* promoter sequences were captured using Apollo Genome Sequence and Annotation Tool (Lewis et al. 2002). Match v1.0-public (BIOBASE Biological Databases, Wolfenbüttel, Germany) was used to search promoter sequences for known transcription factor binding sites using a library of mononucleotide-weighted matrices from TRANSFAC v6.0. Match v1.0-public employs the core- and matrices-matching algorithms published by Quandt et al. (1995). Sequences were interrogated using only high-quality *Drosophila* transcription factor binding sites found in TRANSFAC v6.0, and the software parameters were adjusted to minimize the sum of false positives and false negatives. The number of Hairy binding sites found in target gene promoters was tallied (excluding “hits” to AT-rich regions [assigned to CF2-II, BRC-Z1, and BRC-Z4] that were ubiquitous in both the target and nontarget sequence under analysis). Using the Hairy site closest to transcription start site, the composition of transcription factor binding sites adjacent to (within 500 bp of) the Hairy site was assessed. This was also performed for non-Hairy targets selected because they contained one or more core C-box sequences. Matrices were compared that matched percentages of known Hairy targets (*egh2, egh3, prd1, ac1, and stg1*) to C-box–containing nontargets.

#### Chromosomes.

Wild-type or pstg βE4.9 third instar larval salivary gland polytene chromosomes were prepared and stained for endogenous proteins essentially as described by Andrew and Scott (1994). Antisera used were as follows: rat anti-Hairy polyclonal (1:50 dilution; gift of J. Reinitz; Kosman et al. 1998), mouse anti-Groucho monoclonal (1:40 dilution; gift of C. Delidakis; Delidakis et al. 1991), mouse anti-dCtBP polyclonal (1:100; Poortinga et al. 1998); mouse anti-dSir2 polyclonal (1:20 dilution; Rosenberg and Parkhurst 2002); rabbit anti-β-galactosidase polyclonal (1:1000); donkey antirat Alexa 488 (1:1000 dilution; Molecular Probes, Eugene, Oregon, United States); and goat antimouse Texas Red (1:200; Jackson ImmunoResearch Laboratories, West Grove, Pennsylvania, United States). Chromosomes were viewed on an Olympus (Tokyo, Japan) IX-70 inverted microscope equipped with a 40×/N.A. 1.35 oil immersion objective. Three-dimensional stacks were collected using the DeltaVision softWoRx acquisition software (Applied Precision, Issaquah, Washington, United States), and out-of-focus information was removed using a constrained iterative deconvolution algorithm (Agard et al. 1989).

The insertion site for the pstg β-E4.9 reporter line was performed as described by Pardue and Gall (1975) using DIG-substituted probes according to the protocol of Tautz and Pfeifle (1989).

## Supporting Information

Dataset S1 Complete List of Binding Loci for Hairy in Kc Cells and Embryos As Well As the Cofactors Groucho (Kc Cells), dCtBP (Kc Cells), and dSir2 (Kc Cells) (2.9 MB XLS).

Dataset S2 DamID Primary Binding Data for Hairy in Kc Cells (11.8 MB XLS).

Dataset S3 DamID Primary Binding Data for Hairy in Embryos (11.8 MB XLS).

Dataset S4 DamID Primary Binding Data for Groucho in Kc Cells (11.8 MB XLS).

Dataset S5 DamID Primary Binding Data for dCtBP in Kc Cells (11.8 MB XLS)

Dataset S6 DamID Primary Binding Data for dSir2 in Kc Cells (11.8 MB XLS)

Dataset S7 List of the 155 Target Loci That Recruit Groucho (Duplicates Removed) (230 KB XLS).

Dataset S8 List of the 496 Target Loci That Recruit dCtBP (Duplicates Removed) (276 KB XLS).

Dataset S9 List of the 107 Target Loci That Recruit dSir2 (Duplicates Removed) (44 KB XLS).

## Abbreviations

*ac*: *achaete*
bHLH: basic Helix-Loop-Helix
CtBP: C-terminal binding protein
Dam: DNA adenine methyltransferase
dCtBP: *Drosophila* C-terminal binding protein
DGC: *Drosophila* Gene Collection
dSir2: *Drosophila* silent information regulator 2
*egh*: *egghead*
EMSA: electromobility shift assay
*ftz*: *fushi tarazu*
HES: Hairy/Enhancer of split/Deadpan
*hkb*: *huckebein*
NAD: nicotinamide adenine dinucleotide
*prd*: *paired*
Sir2: silent information regulator 2
*stg*: *string*

## References

[pbio-0020178-Agard1] Agard DA, Hiraoka Y, Shaw P, Sedat JW (1989). Fluorescence microscopy in three dimensions. Methods Cell Biol.

[pbio-0020178-Andersen1] Andersen AS, Hansen PH, Schäffer L, Kristensen C (2000). A new secreted insect protein belonging to the immunoglobulin superfamily binds insulin and related peptides and inhibits their activities. J Biol Chem.

[pbio-0020178-Andrew1] Andrew DJ, Scott M (1994). Immunological methods for mapping protein distribution on polytene chromosomes. In: Goldstein LSB, Fyrberg, EA, editors. *Drosophila melanogaster* Practical uses in cell and molecular biology.

[pbio-0020178-Aronson1] Aronson BD, Fisher AL, Blechman K, Caudy M, Gergen JP (1997). Groucho-dependent and -independent repression activities of Runt domain proteins. Mol Cell Biol.

[pbio-0020178-Baker1] Baker DA, Mille-Baker B, Wainwright SM, Ish-Horowicz D, Dibb NJ (2001). Mae mediates MAP kinase phosphorylation of Ets transcription factors in *Drosophila*. Nature.

[pbio-0020178-Baldi1] Baldi P, Long AD (2001). A Bayesian framework for the analysis of microarray expression data: Regularized t-test and statistical inferences of gene changes. Bioinformatics.

[pbio-0020178-Barolo1] Barolo S, Levine M (1997). *hairy* mediates dominant repression in the *Drosophila* embryo. EMBO J.

[pbio-0020178-Baumgartner1] Baumgartner S, Noll M (1990). Network of interactions among pair-rule genes regulating paired expression during primordial segmentation of *Drosophila*. Mech Dev.

[pbio-0020178-Beitel1] Beitel GJ, Krasnow MA (2000). Genetic control of epithelial tube size in the *Drosophila* tracheal system. Development.

[pbio-0020178-Bone1] Bone JR, Roth SY (2001). Corepressor proteins and control of transcription in yeast. Curr Top Microbiol Immunol.

[pbio-0020178-Botas1] Botas J, Moscoso del Prado J, García-Bellido A (1982). Gene dose titration analysis in the search for trans-regulatory genes in *Drosophila*. EMBO J.

[pbio-0020178-Brand1] Brand A, Perrimon N (1993). Targeted gene expression as a means of altering cell fates and generating dominant phenotypes. Development.

[pbio-0020178-Brown1] Brown NL, Sattler CA, Paddock SW, Carroll SB (1995). Hairy and Emc negatively regulate morphogenetic furrow progression in the *Drosophila* eye. Cell.

[pbio-0020178-Carroll1] Carroll SB, Scott MP (1986). Zygotically active genes that affect the spatial expression of the *fushi tarazu* segmentation gene during early *Drosophila* embryogenesis. Cell.

[pbio-0020178-Cavallo1] Cavallo RA, Cox RT, Moline MM, Roose J, Polevoy GA (1998). *Drosophila* Tcf and Groucho interact to repress Wingless signalling activity. Nature.

[pbio-0020178-Chen1] Chen G, Courey AJ (2000). Groucho/TLE family proteins and transcriptional repression. Gene.

[pbio-0020178-Chen2] Chen G, Fernandez J, Mische S, Courey AJ (1999). A functional interaction between the histone deacetylase Rpd3 and the corepressor groucho in *Drosophila* development. Genes Dev.

[pbio-0020178-Chinnadurai1] Chinnadurai G (2002a). CtBP, an unconventional transcriptional corepressor in development and oncogenesis. Mol Cell.

[pbio-0020178-Chinnadurai2] Chinnadurai G (2002b). CtBP family proteins: More than transcriptional corepressors. BioEssays.

[pbio-0020178-Courey1] Courey AJ, Jia S (2001). Transcriptional repression: The long and the short of it. Genes Dev.

[pbio-0020178-Dang1] Dang CV, Lewis BC, Dolde C, Dang G, Shim H (1997). Oncogenes in tumor metabolism, tumorigenesis, and apoptosis. J Bioenerg Biomemb.

[pbio-0020178-Davis1] Davis RL, Turner DL (2001). Vertebrate hairy and Enhancer of split related proteins: Transcriptional repressors regulating cellular differentiation and embryonic patterning. Oncogene.

[pbio-0020178-Delidakis1] Delidakis C, Preiss A, Hartley DA, Artavanis-Tsakonas S (1991). Two genetically and molecularly distinct functions involved in early neurogenesis reside within the *Enhancer of split* locus of Drosophila melanogaster. Genetics.

[pbio-0020178-Denu1] Denu JM (2003). Linking chromatin function with metabolic networks: Sir2 family of NAD(+)-dependent deacetylases. Trends Biochem Sci.

[pbio-0020178-Dequier1] Dequier E, Souid S, Pal M, Maroy P, Lepesant JA (2001). Top-DER- and Dpp-dependent requirements for the *Drosophila fos/kayak* gene in follicular epithelium morphogenesis. Mech Dev.

[pbio-0020178-Dobens1] Dobens LL, Martin-Blanco E, Martinez-Arias A, Kafatos FC, Raftery LA (2001). *Drosophila* puckered regulates Fos/Jun levels during follicle cell morphogenesis. Development.

[pbio-0020178-Dubnicoff1] Dubnicoff T, Valentine SA, Chen G, Shi T, Lengyel JA (1997). Conversion of dorsal from an activator to a repressor by the global corepressor Groucho. Genes Dev.

[pbio-0020178-Ebner1] Ebner A, Kiefer FN, Ribeiro C, Petit V, Nussbaumer U (2002). Tracheal development in Drosophila melanogaster as a model system for studying the development of a branched organ. Gene.

[pbio-0020178-Edgar1] Edgar BA, Weir MP, Schubiger G, Kornberg T (1986). Repression and turnover pattern *fushi tarazu* RNA in the early *Drosophila* embryo. Cell.

[pbio-0020178-Fisher1] Fisher A, Caudy M (1998a). The function of hairy-related bHLH repressor proteins in cell fate decisions. Bioessays.

[pbio-0020178-Fisher2] Fisher AL, Caudy M (1998b). Groucho proteins: Transcriptional corepressors for specific subsets of DNA-binding transcription factors in vertebrates and invertebrates. Genes Dev.

[pbio-0020178-Fisher3] Fisher AL, Ohsako S, Caudy M (1996). The WRPW motif of the Hairy-related basic helix-loop-helix repressor proteins acts as a 4-amino acid transcription repression and protein–protein interaction domain. Mol Cell Biol.

[pbio-0020178-Fjeld1] Fjeld CC, Birdsong WT, Goodman RH (2003). Differential binding of NAD+ and NADH allows the transcriptional corepressor carboxyl-terminal binding protein to serve as a metabolic sensor. Proc Natl Acad Sci U S A.

[pbio-0020178-Francastel1] Francastel C, Schubeler D, Martin DI, Groudine M (2000). Nuclear compartmentalization and gene activity. Nat Rev Mol Cell Biol.

[pbio-0020178-Fulco1] Fulco M, Schiltz RL, Iezzi S, King MT, Zhao P (2003). Sir2 regulates skeletal muscle differentiation as a potential sensor of the redox state. Mol Cell.

[pbio-0020178-Gallant1] Gallant P, Shiio Y, Cheng PF, Parkhurst SM, Eisenman RN (1996). Myc and Max homologs in *Drosophila*. Science.

[pbio-0020178-Garbe1] Garbe JC, Yang E, Fristrom JW (1993). IMP-L2: An essential secreted immunoglobulin family member implicated in neural and ectodermal development in *Drosophila*. Development.

[pbio-0020178-Gaston1] Gaston K, Jayaraman PS (2003). Transcriptional repression in eukaryotes: Repressors and repression mechanisms. Cell Mol Life Sci.

[pbio-0020178-Giot1] Giot L, Bader JS, Brouwer C, Chaudhuri A, Kuang B (2003). A protein interaction map of Drosophila melanogaster. Science.

[pbio-0020178-Goode1] Goode S, Melnick M, Chou T-B, Perrimon N (1996). The neurogenic genes *egghead* and *brainiac* define a novel signaling pathway essential for epithelial morphogenesis during *Drosophila* oogenesis. Development.

[pbio-0020178-Gottschling1] Gottschling DE (2000). Gene silencing: Two faces of SIR2. Curr Biol.

[pbio-0020178-Gray1] Gray S, Levine M (1996). Transcriptional repression in development. Curr Op Cell Biol.

[pbio-0020178-Gutjahr1] Gutjahr T, Frei E, Noll M (1993). Complex regulation of early *paired* expression: Initial activation by gap genes and pattern modulation by pair-rule genes. Development.

[pbio-0020178-Hanna-Rose1] Hanna-Rose W, Hansen U (1996). Active repression mechanisms of eukaryotic transcription repressors. Trends Genet.

[pbio-0020178-Henikoff1] Henikoff S, Ahmad K, Platero JS, van Steensel B (2000). Heterochromatic deposition of centromeric histone H3-like proteins. Proc Natl Acad Sci U S A.

[pbio-0020178-Herschbach1] Herschbach BM, Johnson AD (1993). Transcriptional repression in eukaryotes. Annu Rev Cell Biol.

[pbio-0020178-Hill1] Hill RJ, Mott MR (2000). Native polytene chromosomes of Drosophila melanogaster for light and electron microscopic observation of the conformation and distribution of molecules. Methods Mol Biol.

[pbio-0020178-Hill2] Hill RJ, Mott MR, Steffensen DM (1987). The preparation of polytene chromosomes for localization of nucleic acid sequences, proteins, and chromatin conformation. Int Rev Cytol.

[pbio-0020178-Howard1] Howard K, Ingham P (1986). Regulatory interactions between the segmentation genes *fushi tarazu, hairy* and *engrailed* in the *Drosophila* blastoderm. Cell.

[pbio-0020178-Imai1] Imai S, Armstrong CM, Kaeberlein M, Guarente L (2000). Transcriptional silencing and longevity protein Sir2 is an NAD-dependent histone deacetylase. Nature.

[pbio-0020178-Ingham1] Ingham PW, Pinchin SM, Howard KR, Ish-Horowicz D (1985). Genetic analysis of the *hairy* locus in Drosophila melanogaster. Genetics.

[pbio-0020178-Ish-Horowicz1] Ish-Horowicz D, Pinchin SM (1987). Pattern abnormalities induced by ectopic expression of the *Drosophila* gene *hairy* are associated with repression of *fushi tarazu* transcription. Cell.

[pbio-0020178-James1] James KE, Dorman JB, Berg CA (2002). Mosaic analyses reveal the function of *Drosophila* Ras in embryonic dorsoventral patterning and dorsal follicle cell morphogenesis. Development.

[pbio-0020178-Jennings1] Jennings BH, Tyler DM, Bray SJ (1999). Target specificities of *Drosophila enhancer of split* basic helix-loop-helix proteins. Mol Cell Biol.

[pbio-0020178-Jepsen1] Jepsen K, Rosenfeld MG (2002). Biological roles and mechanistic actions of co-repressor complexes. J Cell Sci.

[pbio-0020178-Jimenez1] Jiménez G, Paroush Z, Ish-Horowicz D (1997). Groucho acts as a corepressor for a subset of negative regulators, including Hairy and Engrailed. Genes Dev.

[pbio-0020178-Johnston1] Johnston LA, Gallant P (2002). Control of growth and organ size in *Drosophila*. Bioessays.

[pbio-0020178-Kawamura1] Kawamura K, Shibata T, Saget O, Peel D, Bryant PJ (1999). A new family of growth factors produced by the fat body and active on *Drosophila* imaginal disc cells. Development.

[pbio-0020178-Kosman1] Kosman D, Small S, Reinitz J (1998). Rapid preparation of a panel of polyclonal antibodies to *Drosophila* segmentation proteins. Dev Genes Evol.

[pbio-0020178-Kumar1] Kumar V, Carlson JE, Ohgi KA, Edwards TA, Rose DW (2002). Transcription corepressor CtBP is an NAD^+^-regulated dehydrogenase. Mol Cell.

[pbio-0020178-Lane1] Lane ME, Sauer K, Wallace K, Jan YN, Lehner CF (1996). Dacapo, a cyclin-dependent kinase inhibitor, stops cell proliferation during *Drosophila* development. Cell.

[pbio-0020178-Lawrence1] Lawrence PA (1992). The making of a fly: The genetics of animal design.

[pbio-0020178-Lehman1] Lehman DA, Patterson B, Johnston LA, Balzer T, Britton JS (1999). Cis-regulatory elements of the mitotic regulator, *string/Cdc25*. Development.

[pbio-0020178-Leitch1] Leitch AR (2000). Higher levels of organization in the interphase nucleus of cycling and differentiated cells. Microbiol Mol Biol Rev.

[pbio-0020178-Lewis1] Lewis SE, Searle SM, Harris N, Gibson M, Lyer V (2002). Apollo: A sequence annotation editor. Genome Biol.

[pbio-0020178-Mannervik1] Mannervik M (2001). Corepressor proteins in *Drosophila* development. Curr Top Microbiol Immunol.

[pbio-0020178-Mannervik2] Mannervik M, Levine M (1999). The Rpd3 histone deacetylase is required for segmentation of the *Drosophila* embryo. Proc Natl Acad Sci U S A.

[pbio-0020178-Mannervik3] Mannervik M, Nibu Y, Zhang H, Levine M (1999). Transcriptional coregulators in development. Science.

[pbio-0020178-Meyer1] Meyer CA, Kramer I, Dittrich R, Marzodko S, Emmerich J (2002). *Drosophila* p27Dacapo expression during embryogenesis is controlled by a complex regulatory region independent of cell cycle progression. Development.

[pbio-0020178-Montagne1] Montagne J, Radimerski T, Thomas G (2001). Insulin signaling: Lessons from the *Drosophila* tuberous sclerosis complex, a tumor suppressor. Sci STKE.

[pbio-0020178-Mullins1] Mullins MC, Rio DC, Rubin GM (1989). *cis*-acting DNA sequence requirements for P-element transposition. Genes Dev.

[pbio-0020178-Myat1] Myat MM, Andrew DJ (2002). Epithelial tube morphology is determined by the polarized growth and delivery of apical membrane. Cell.

[pbio-0020178-Newman1] Newman BL, Lundblad JR, Chen Y, Smolik SM (2002). A *Drosophila* homologue of *sir2* modifies position-effect variegation but does not affect life span. Genetics.

[pbio-0020178-Nibu1] Nibu Y, Zhang H, Levine M (1998a). Interaction of short-range repressors with *Drosophila* CtBP in the embryo. Science.

[pbio-0020178-Nibu2] Nibu Y, Zhang H, Bajor E, Barolo S, Small S (1998b). dCtBP mediates transcriptional repression by Knirps, Kruppel and Snail in the *Drosophila* embryo. EMBO J.

[pbio-0020178-Oellers1] Oellers N, Dehio M, Knust E (1994). bHLH proteins encoded by the *Enhancer of split* complex of *Drosophila* negatively interfere with transcriptional activation mediated by proneural genes. Mol Gen Genet.

[pbio-0020178-Ogata1] Ogata K, Sato K, Tahirov TH, Tahirov T (2003). Eukaryotic transcriptional regulatory complexes: cooperativity from near and afar. Curr Opin Struct Biol.

[pbio-0020178-Ohsako1] Ohsako S, Hyer J, Panganiban G, Oliver I, Caudy M (1994). *hairy* function as a DNA-binding helix-loop-helix repressor of *Drosophila* sensory organ formation. Genes Dev.

[pbio-0020178-Orian1] Orian A, van Steensel B, Delrow J, Bussemaker HJ, Li L (2003). Genomic binding by the *Drosophila* Myc, Max, Mad/Mnt transcription factor network. Genes Dev.

[pbio-0020178-Pardue1] Pardue ML, Gall JG (1975). Nucleic acid hybridization to the DNA of cytological preparations. Methods Cell Biol.

[pbio-0020178-Parkhurst1] Parkhurst SM (1998). Groucho: Making its Marx as a transcriptional corepressor. Trends Genet.

[pbio-0020178-Parkhurst2] Parkhurst SM, Bopp D, Ish-Horowicz D (1990). X:A ratio is transduced by helix-loop-helix proteins in *Drosophila*. Cell.

[pbio-0020178-Parks1] Parks S, Wieschaus E (1991). The *Drosophila* gastrulation gene *concertina* encodes a G alpha-like protein. Cell.

[pbio-0020178-Paroush1] Paroush Z, Finley RL, Kidd T, Wainwright SM, Ingham PW (1994). Groucho is required for *Drosophila* neurogenesis, segmentation and sex-determination, and interacts directly with Hairy-related bHLH proteins. Cell.

[pbio-0020178-Phippen1] Phippen TM, Sweigart AL, Moniwa M, Krumm A, Davie JR (2000). *Drosophila* CtBP functions as a context-dependent transcriptional cofactor and interferes with both Mad and Groucho transcriptional repression. J Biol Chem.

[pbio-0020178-Pile1] Pile LA, Wassarman DA (2000). Chromosomal localization links the SIN3-RPD3 complex to the regulation of chromatin condensation, histone acetylation and gene expression. EMBO J.

[pbio-0020178-Pile2] Pile LA, Wassarman DA (2002). Localizing transcription factors on chromatin by immunofluorescence. Methods.

[pbio-0020178-Poortinga1] Poortinga G, Watanabe M, Parkhurst SM (1998). *Drosophila* CtBP: A Hairy-interacting protein required for embryonic segmentation and Hairy-mediated transcriptional repression. EMBO J.

[pbio-0020178-Quandt1] Quandt K, Frech K, Karas H, Wingender E, Werner T (1995). MatInd and MatInspector: New fast and versatile tools for detection of consensus matches in nucleotide sequence data. Nucleic Acids Res.

[pbio-0020178-Ramet1] Ramet M, Lanot R, Zachary D, Manfruelli P (2002). JNK signaling pathway is required for efficient wound healing in *Drosophila*. Dev Biol.

[pbio-0020178-Riesgo-Escovar1] Riesgo-Escovar JR, Hafen E (1997). *Drosophila* Jun kinase regulates expression of decapentaplegic via the ETS-domain protein Aop and the AP-1 transcription factor DJun during dorsal closure. Genes Dev.

[pbio-0020178-Roose1] Roose J, Molenaar M, Peterson J, Hurenkamp J, Brantjes H (1998). The Xenopus Wnt effector XTcf-3 interacts with Groucho-related transcriptional repressors. Nature.

[pbio-0020178-Rorth1] Rørth P (1998). Gal4 in the *Drosophila* female germline. Mech Dev.

[pbio-0020178-Rosenberg1] Rosenberg MI, Parkhurst SM (2002). *Drosophila Sir2* is required for heterochromatic silencing and by euchromatic Hairy/E(Spl) bHLH repressors in segmentation and sex determination. Cell.

[pbio-0020178-Rubin1] Rubin GM, Hong L, Brokstein P, Evans-Holm M, Frise E (2000). A *Drosophila* complementary DNA resource. Science.

[pbio-0020178-Rushlow1] Rushlow CA, Hogan A, Pinchin SM, Howe KM, Lardelli MT (1989). The Drosophila hairy protein acts in both segmentation and bristle patterning and shows homology to N-myc. EMBO J.

[pbio-0020178-Sasai1] Sasai Y, Kageyama R, Tagawa Y, Shigemoto R, Nakanishi S (1992). Two mammalian helix loop helix factors structurally related to Drosophila hairy and *Enhancer-of-split*. Genes Dev.

[pbio-0020178-Shi1] Shi Y, Sawada J, Sui G, Affar EB, Whetstine JR (2003). Coordinated histone modifications mediated by a CtBP co-repressor complex. Nature.

[pbio-0020178-Spellman1] Spellman PT, Rubin GM (2002). Evidence for large domains of similarly expressed genes in the *Drosophila* genome. J Biol.

[pbio-0020178-Spradling1] Spradling AC (1986). P element–mediated transformation. In: Roberts DB, editor. *Drosophila* A practical approach.

[pbio-0020178-Sundqvist1] Sundqvist A, Sollerbrant K, Svensson C (1998). The carboxy-terminal region of the adenovirus E1A activates transcription through targeting of a C-terminal binding protein–histone deacetylase complex. FEBS Lett.

[pbio-0020178-Takata1] Takata T, Ishikawa F (2003). Human Sir2-related protein SIRT1 associates with the bHLH repressors HES1 and HEY2 and is involved in HES1- and HEY2-mediated transcriptional repression. Biochem Biophys Res Commun.

[pbio-0020178-Tapon1] Tapon N, Moberg KH, Hariharan IK (2001). The coupling of cell growth to the cell cycle. Curr Opin Cell Biol.

[pbio-0020178-Tautz1] Tautz D, Pfeifle C (1989). A nonradioactive in situ hybridization method for the localization of specific RNAs in *Drosophila* embryos reveals translational control of the segmentation gene hunchback. Chromosoma.

[pbio-0020178-Tietze1] Tietze K, Oellers N, Knust E (1992). *Enhancer of split^D^* a dominant mutation of *Drosophila* and its use in the study of functional domains of a helix-loop-helix protein. Proc Natl Acad Sci U S A.

[pbio-0020178-Turner1] Turner J, Crossley M (2001). The CtBP family: Enigmatic and enzymatic transcriptional co-repressors. Bioessays.

[pbio-0020178-Urnov1] Urnov FD, Wolffe AP, Guschin D (2001). Molecular mechanisms of corepressor function. Curr Top Microbiol Immunol.

[pbio-0020178-Van1] Van Doren M, Bailey AM, Esnayra J, Ede K, Posakony JW (1994). Negative regulation of proneural gene activity: *hairy* is a direct transcriptional repressor of *achaete*. Genes Dev.

[pbio-0020178-van2] van Steensel B, Henikoff S (2000). Identification of in vivo DNA targets of chromatin proteins using tethered dam methyltransferase. Nat Biotechnol.

[pbio-0020178-van3] van Steensel B, Delrow J, Henikoff S (2001). Chromatin profiling using targeted DNA adenine methyltransferase. Nat Genet.

[pbio-0020178-Wieschaus1] Wieschaus E, Nüsslein-Volhard C (1986). Looking at embryos. In: Roberts DB, editor. *Drosophila* A practical approach.

[pbio-0020178-Yamada1] Yamada K, Kawata H, Shou Z, Mizutani T, Noguchi T (2003). Insulin induces the expression of the SHARP-2/Stra13/DEC1 gene via a phosphoinositide 3-kinase pathway. J Biol Chem.

[pbio-0020178-Yu1] Yu Y, Pick L (1995). Non-periodic cues generate seven ftz stripes in the *Drosophila* embryo. Mech Dev.

[pbio-0020178-Zhang1] Zhang H, Levine M (1999). Groucho and dCtBP mediate separate pathways of transcriptional repression in the *Drosophila* embryo. Proc Natl Acad Sci U S A.

[pbio-0020178-Ziegler1] Ziegler M (2000). New functions of a long-known molecule. Emerging roles of NAD in cellular signaling. Eur J Biochem.

